# Plasma Calprotectin Levels Associate with Suspected Metabolic-Associated Fatty Liver Disease and All-Cause Mortality in the General Population

**DOI:** 10.3390/ijms232415708

**Published:** 2022-12-11

**Authors:** Arno R. Bourgonje, Eline H. van den Berg, Lyanne M. Kieneker, Tom Nilsen, Clara Hidden, Stephan J. L. Bakker, Hans Blokzijl, Robin P. F. Dullaart, Harry van Goor, Amaal E. Abdulle

**Affiliations:** 1 Department of Gastroenterology and Hepatology, University of Groningen, University Medical Center Groningen, 9713 GZ Groningen, The Netherlands; 2 Department of Internal Medicine, Division of Nephrology, University of Groningen, University Medical Center Groningen, 9713 GZ Groningen, The Netherlands; 3 Gentian AS, 1596 Moss, Norway; 4 Department of Endocrinology, University of Groningen, University Medical Center Groningen, 9713 GZ Groningen, The Netherlands; 5 Department of Pathology and Medical Biology, University of Groningen, University Medical Center Groningen, 9713 GZ Groningen, The Netherlands; 6 Department Internal Medicine, Division of Vascular Medicine, University of Groningen, University Medical Center Groningen, 9713 GZ Groningen, The Netherlands

**Keywords:** metabolic-associated fatty liver disease (MAFLD), calprotectin, inflammation, fatty liver index, hepatic steatosis index

## Abstract

Metabolic-associated fatty liver disease (MAFLD) is characterized by hepatic steatosis, metabolic dysregulation, and neutrophilic inflammation. In this study, we hypothesized that systemic levels of plasma calprotectin, as a biomarker of neutrophilic inflammation, may be associated with suspected MAFLD. Plasma calprotectin levels were measured in subjects (*n* = 5446) participating in the Prevention of Renal and Vascular ENd-stage Disease (PREVEND) cohort study. Suspected MAFLD was defined by the fatty liver index (FLI ≥ 60) and hepatic steatosis index (HSI ≥ 36) as proxies. Plasma calprotectin levels were significantly higher in subjects with FLI ≥ 60 (0.57 [IQR: 0.42–0.79] mg/L, *n* = 1592) (*p* < 0.001) compared to subjects with FLI < 60 (0.46 [0.34–0.65] mg/L, *n* = 3854). Multivariable logistic regression analyses revealed that plasma calprotectin levels were significantly associated with suspected MAFLD (FLI ≥ 60), even after adjustment for potential confounding factors, including current smoking, alcohol consumption, hypertension, diabetes, cardiovascular diseases, insulin resistance (HOMA-IR), hs-CRP, eGFR, and total cholesterol levels (OR 1.19 [95% CI: 1.06–1.33], *p* = 0.003). Interaction analyses revealed significant effect modifications for the association between plasma calprotectin and suspected MAFLD by BMI (*p* < 0.001) and hypertension (*p* = 0.003), with the strongest associations in subjects with normal BMI and without hypertension. Prospectively, plasma calprotectin levels were significantly associated with all-cause mortality after adjustment for potential confounding factors, particularly in subjects without suspected MAFLD (FLI < 60) (hazard ratio (HR) per doubling: 1.34 (1.05–1.72), *p* < 0.05). In conclusion, higher plasma calprotectin levels are associated with suspected MAFLD and with the risk of all-cause mortality, the latter especially in subjects without suspected MAFLD.

## 1. Introduction

Metabolic-associated fatty liver disease (MAFLD), also referred to as non-alcoholic fatty liver disease (NAFLD), represents a spectrum of events characterized by excessive lipid accumulation in hepatocytes ranging from non-alcoholic steatohepatitis (NASH) to fibrosis, cirrhosis, and in some cases, hepatocellular carcinoma [1]. MAFLD is characterized by hepatic steatosis combined with obesity, type 2 diabetes (T2D), evidence of metabolic dysregulation, or a combination of these factors [1,2,3]. The global incidence of MAFLD is steadily rising, and it is emerging as the most prevalent chronic liver disease in Western countries. In population-based epidemiological studies, a suspicion of MAFLD is frequently estimated by calculating indices as proxies of the disease based on clinical and biochemical variables. Two commonly used indices include the Fatty Liver Index (FLI) and the Hepatic Steatosis Index (HSI), which are considered as potential predictors of suspected MAFLD and are based on classical risk factors of the disease, including body-mass index (BMI), waist circumference, and plasma levels of triglycerides, gamma-glutamyltransferase, and liver transaminases [4,5].

Accumulating evidence points to a significant role of inflammation as a pathophysiological driver of MAFLD progression [6]. In the development of MAFLD, hepatocytes suffer from cellular stress as a result of lipotoxicity, resulting in disrupted mitochondrial and peroxisomal fatty acid oxidation and endoplasmic reticulum stress. Concomitantly, inflammatory signaling cascades such as those mediated by the NF-κB transcription factor become activated in the liver, leading to the release of a number of inflammatory (e.g., cytokines such as IL-1, IL-6, and TNF-α) and oxidative (e.g., reactive oxygen species, ROS) mediators [7,8]. Consequently, hepatocellular injury and -death occurs, which contributes to the development of MAFLD but also further aggravates pro-inflammatory signaling. Neutrophilic granulocytes play a pivotal role in the initiation and progression of these pro-inflammatory cascades, and act by the production of ROS, defensins, neutrophil extracellular traps (NETs), and by phagocytosis [9]. Neutrophils are among the first immune cells to invade the liver upon MAFLD development and attract other types of immune cells into the histological infiltrate. The role of neutrophils in MAFLD is exemplified by studies showing strong correlations between neutrophil-to-lymphocyte ratios (NLR), as well as increases in circulating levels of neutrophil-derived products such as myeloperoxidase (MPO), NETs, and proteinase-3 (PR3) in MAFLD [10,11,12].

Calprotectin is a 36 kDa calcium- and zinc-binding protein dimer (also known as the S100A8/S100A9 complex) that is present in the cytosol of neutrophilic granulocytes [13]. Upon inflammation, calprotectin is actively secreted by neutrophils as part of their stress response, executing its pro-inflammatory functions as an alarmin and functioning as antimicrobial components as part of NETs [14,15]. Furthermore, it is considered as an acute-phase protein and has been associated with a number of inflammatory conditions, including rheumatoid arthritis, psoriasis, as well as cardiovascular diseases [16,17,18]. Of note, fecal calprotectin is clinically used as a reliable biomarker of active intestinal inflammation in patients with inflammatory bowel disease (IBD) [19]. Given the role of hepatic infiltration of neutrophils and systemic inflammation in MAFLD, circulating calprotectin levels may also reflect the implication of neutrophil inflammation in the pathogenesis of MAFLD. However, only a limited number of (pre-) clinical studies have been performed examining calprotectin as biomarker in subjects with MAFLD or NASH, and conflicting results have been reported [20,21,22].

Since neutrophilic inflammation is known to play a role in the pathophysiology of MAFLD [23], we hypothesized that plasma calprotectin levels might have merit as a biomarker for suspected MAFLD. Therefore, we determined plasma calprotectin levels in serum from 5446 participants of a large population-based cohort study. In this study, we aimed to investigate whether plasma calprotectin is associated with suspected MAFLD in subjects derived from the general population. Secondly, we aimed to determine associations between baseline plasma calprotectin levels and all-cause mortality in subjects with MAFLD during long-term follow-up.

## 2. Results

### 2.1. Baseline Characteristics of the Study Population

Baseline demographic, clinical, and laboratory characteristics of the study population are presented in Table 1. In total, 5446 study participants were included, of whom 1592 subjects (29.2%) had a FLI ≥ 60 and 3854 subjects (70.8%) a FLI < 60. Participants who were classified as FLI ≥ 60 were older (*p* < 0.001) and were more often male (*p* < 0.001). In addition, participants with a FLI ≥ 60 more frequently had metabolic syndrome (MetS), a history of diabetes, and a history of cardiovascular disease (all *p* < 0.001) and more frequently used antihypertensive, antidiabetic, and lipid-lowering drugs (all *p* < 0.001). Furthermore, anthropometric measurements (i.e., BMI, waist circumference, waist/hip ratio) revealed higher values for subjects with a FLI ≥ 60 as well as cholesterol and liver transaminase levels, which were all increased in subjects with a FLI ≥ 60 (all *p* < 0.001). Plasma calprotectin levels were significantly elevated in subjects with a FLI ≥ 60 compared to those with a FLI < 60 (0.57 [0.42–0.79] vs. 0.46 [0.34–0.65], *p* < 0.001). Similar results were obtained when participants with an HSI < 36 and HSI ≥ 36 were compared for baseline demographic, clinical and laboratory characteristics (Supplementary Table S1).

Subsequently, baseline demographic, clinical and laboratory characteristics of study participants were categorized according to tertiles of plasma calprotectin levels, as presented in Supplementary Table S2. Participants within the highest tertile of plasma calprotectin levels were older (*p* < 0.001) and more likely to be male (*p* < 0.001), and more often presented with comorbidities such as MetS, T2DM, and a history of cardiovascular disease. Similarly, participants within the highest tertile more often used antihypertensive and lipid-lowering drugs and had higher values from anthropometric measurements (BMI, waist circumference, and waist/hip ratio), as well as higher levels of total cholesterol, triglycerides, glucose, insulin, and HOMA-IR (all *p* < 0.001) compared to participants within the 1st and 2nd tertiles of plasma calprotectin levels.

### 2.2. Associations between Plasma Calprotectin Levels and Suspected MAFLD

**Figure 1:**
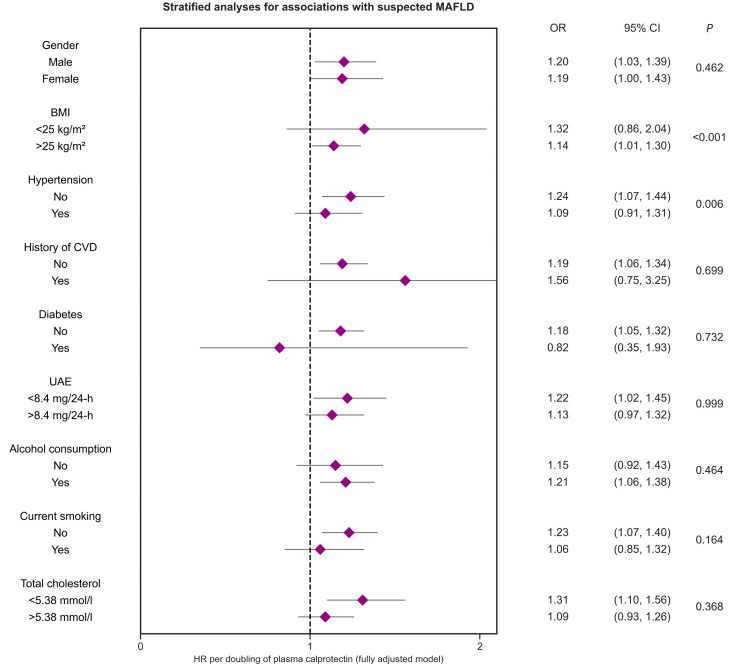
Stratified analyses for the association between plasma calprotectin levels and suspected MALFD (FLI ≥ 60) across various subgroups. Odds ratios (ORs) with corresponding 95% confidence intervals derived from multivariable logistic regression analyses are shown. *p*-values indicate *p*-values for interaction terms. First, we tested for potential effect modification for the variables shown, and subsequently performed stratified analyses. ORs demonstrate almost consistently positive associations between plasma calprotectin levels and the risk of all-cause mortality in all analyzed subgroups. ORs were adjusted for potential confounding factors, including all factors incorporated in *Model 4* (Table 2). Abbreviations: CI, confidence interval; BMI, body mass index; CVD, cardiovascular disease; HR, hazard ratio; UAE, urinary albumin excretion.

Logistic regression analyses were performed to establish the extent to which plasma levels of calprotectin were associated with suspected MAFLD as defined by a FLI ≥ 60 (Table 2). Indeed, higher levels of plasma calprotectin (^2^log-transformed, per doubling of concentration) were significantly associated with suspected MAFLD (OR 1.68 [1.56–1.82], *p* < 0.001), which did not materially change after adjustment for age and sex (*Model 2*, OR 1.58 [1.45–1.71], *p* < 0.001). Subsequently, we adjusted for the potential confounding effects of current smoking, alcohol consumption, history of diabetes, history of cardiovascular disease, and the presence of hypertension, after which the association between plasma calprotectin and suspected MAFLD remained significant (*Model 3*, OR 1.51 [1.38–1.64], *p* < 0.001). After additional adjustment for total cholesterol levels, renal function (eGFR), insulin resistance (HOMA-IR), and hs-CRP levels, the association was still statistically significant (*Model 4*, OR 1.19 [1.06–1.33], *p* = 0.003). Similar results were obtained when the HSI instead of the FLI was used as a proxy for suspected MAFLD, demonstrating a statistically significant association after adjustment for the same covariates (*Model 4*, OR 1.24 [1.12–1.38], *p* < 0.001) (Supplementary Table S3). Subsequently, we tested for potential effect-modification across various subgroups and performed stratified analyses, revealing significant effect-modification for BMI (*p* < 0.001) and the presence of hypertension (*p* = 0.006) (Figure 1, Supplementary Table S4). Corresponding ORs were higher for subjects with lower BMI (<25 kg/m^2^) and subjects without hypertension. Similar results were obtained when the HSI score was used as proxy for suspected MAFLD, showing significant effect-modification for BMI (*p* < 0.001), the presence of hypertension (*p* = 0.030), and additionally for sex (*p* = 0.013), with higher ORs for subjects with lower BMI (<25 kg/m^2^), subjects without hypertension, and women (Supplementary Table S5).

### 2.3. Plasma Calprotectin Levels and Risk of All-Cause Mortality in Subjects with and without Suspected MAFLD

**Figure 2:**
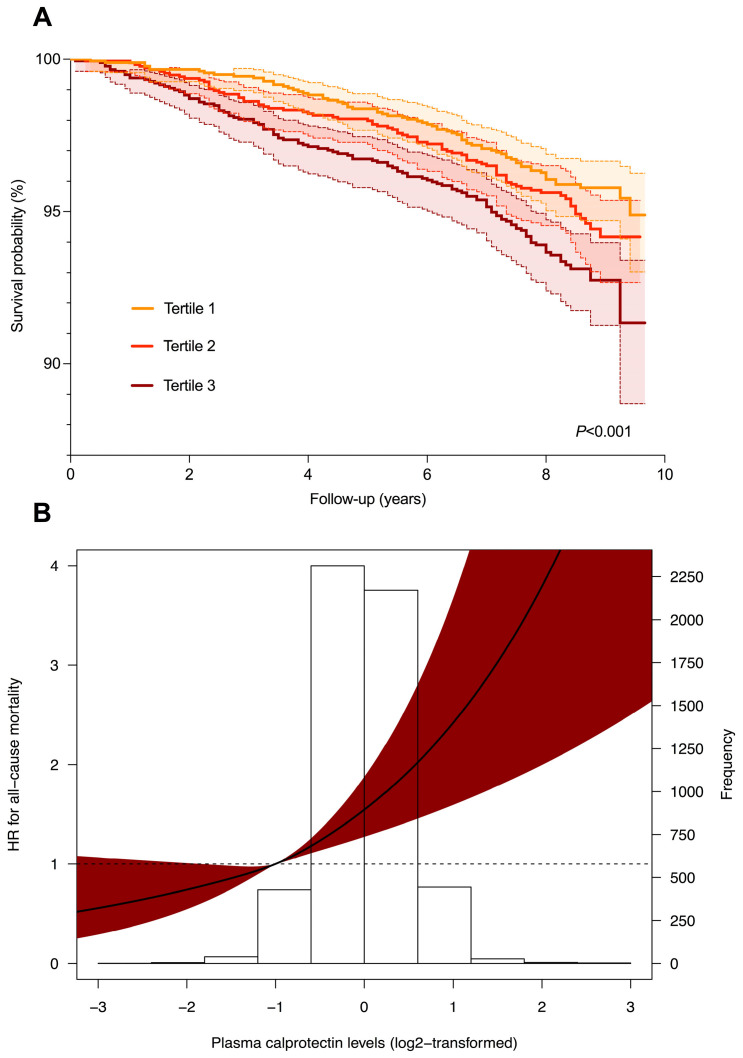
Plasma calprotectin levels are associated with the risk of all-cause mortality in the general population. (**A**) Kaplan–Meier survival curves for tertiles of plasma calprotectin levels, showing survival distributions with 95% confidence intervals based on the outcome of all-cause mortality. The highest rate of all-cause mortality was observed in the highest tertile of plasma calprotectin levels (log-rank test, *p* < 0.001). (**B**) Restricted cubic spline (RCS) regression demonstrating the relationship between plasma calprotectin levels and the risk of all-cause mortality. Estimates were derived from the Cox proportional hazards regression model, while RCS was based on three knots set at the 10th, 50th, and 90th percentiles. A likelihood ratio test for nonlinearity was not statistically significant (*p* = 0.519). The dark-red-shaded area represents the 95% confidence interval. Abbreviations: HR, hazards ratio.

During follow-up, 274 participants (5.0%) died (FLI < 60: 154 (4.0%); FLI ≥ 60: 120 (7.5%)). Using tertiles of plasma calprotectin levels, Kaplan–Meier survival analysis demonstrated significantly differential survival distributions (Figure 2A, *p* < 0.001, log-rank test), especially among subjects without suspected MAFLD (*p* < 0.001, log–rank test), but not solely in subjects with suspected MAFLD (*p* = 0.689, log–rank test). Cox proportional hazards regression analyses demonstrated a significant positive association between ^2^log-transformed plasma calprotectin levels and the risk of all-cause mortality (HR per doubling 1.48 [1.28–1.71], *p* < 0.001, Table 3A). This association remained statistically significant after adjusting for age, sex, history of cardiovascular disease, diabetes, hypertension, current smoking, and alcohol use (*Model 3*, HR per doubling 1.17 [1.00–1.38), *p* = 0.049), but vanished after additional adjustment for HOMA-IR, hs-CRP, eGFR, and total cholesterol levels (*Model 4*, HR per doubling 1.10 [0.91–1.33], *p* = 0.348). Restricted cubic splines demonstrated no significant deviations from linear associations with the risk of all-cause mortality (Figure 2B, *p* = 0.519). When stratifying by the presence of suspected MAFLD, the association between ^2^log-transformed plasma calprotectin levels and the risk of all-cause mortality was statistically significant after adjusting for potential confounding factors in subjects with FLI < 60 (*Model 4*, HR 1.34 [1.05–1.72], *p* = 0.019, Table 3B), while no significant associations were found for subjects with FLI ≥ 60 (Table 3C). When taking the HSI instead of the FLI as proxy for suspected MAFLD, similar results were obtained in Cox proportional hazards regression analyses, i.e., the association between ^2^log-transformed plasma calprotectin levels and the risk of all-cause mortality being statistically significant in subjects with HSI < 36, while being absent in subjects with HSI ≥ 36 (Supplementary Table S6). However, in subjects with HSI < 36, this did not reach formal statistical significance after adjustment for history of cardiovascular disease, diabetes, hypertension, alcohol use, and current smoking (*Model 3*, HR 1.20 (1.00–1.44), *p* = 0.054).

## 3. Discussion

This large-scale population-based study demonstrates that plasma calprotectin levels are increased in subjects with suspected MAFLD, based on an elevated Fatty Liver Index (FLI) and confirmed by an elevated hepatic steatosis index (HSI). Multivariable analyses showed that plasma calprotectin levels were independently associated with suspected MAFLD after adjustment for a number of relevant confounding factors, including comorbidities (history of cardiovascular disease, diabetes, hypertension), smoking, alcohol use, cholesterol levels, insulin resistance, hs-CRP, and renal function. Stratified analyses demonstrated that there were significantly differential associations of plasma calprotectin levels (per doubling) by BMI and the presence of hypertension. Furthermore, plasma calprotectin levels were significantly associated with an increased risk of all-cause mortality in the total study population but after stratifying by suspected MAFLD, especially in subjects without suspected MAFLD (FLI < 60). These results were confirmed through comparable observations when using the HSI instead of the FLI as a proxy for suspected MAFLD. Collectively, our results indicate that plasma calprotectin may be a promising biomarker for the development and/or presence of MAFLD and that plasma calprotectin levels associate with the risk of all-cause mortality, particularly in subjects with a lower probability of having suspected MAFLD (FLI < 60).

Calprotectin is an inflammation-associated protein that is highly expressed and most abundant in neutrophilic granulocytes, which are implicated in the pathophysiology of MAFLD [24,25]. Since neutrophils are the primary determinants of calprotectin production, plasma calprotectin levels may reflect neutrophil involvement in MAFLD pathogenesis. Neutrophils are principally the first type of immune cells responding to inflammatory stimuli from various tissues, including the liver, and further trigger the recruitment and activation of other inflammatory cells, e.g., antigen-presenting cells [26]. It is therefore not surprising that related indices such as the neutrophil-to-lymphocyte ratio (NLR) are closely associated with the severity of MAFLD. For instance, NLR has been shown to correlate with various pathologic changes in MAFLD, including steatosis, hepatocyte degeneration, inflammation, and fibrosis [27,28,29]. Hepatic neutrophil infiltration is a salient feature of MAFLD progression, and several mechanisms have been proposed to explain how these neutrophils may accelerate disease progression. Primarily, neutrophils produce and release ROS, protein-degrading enzymes, and inflammatory cytokines, which inflict damage to hepatocytes and perpetuate inflammation and fibrosis [23]. In this regard, neutrophil-specific proteins such as myeloperoxidase (MPO), neutrophil elastase (NE), lipocalin-2 (LCN2), and neutrophil extracellular traps (NETs) are major effectors and participate in the host defense not only by killing microorganisms but also through feeding into a positive feedback loop by further potentiating neutrophil function in circumstances of sterile inflammation [30]. Although the exact contribution of calprotectin to MAFLD pathogenesis is not well understood, serum levels of S100A8/S100A9, which play a critical role in modulating the inflammatory response by stimulating leukocyte recruitment and inducing cytokine secretion, have previously been shown to be elevated in patients with NASH [14,31]. Similarly, S100A8/S100A9 proteins are upregulated in experimental animal models of NASH, as well as in adipose tissue of patients with NASH [32]. Considering the role of calprotectin in other inflammatory diseases, it has been suggested that it serves a prominent role in innate immunity by amplifying the immune response via damage-associated molecular patterns (DAMPs) in the context of MAFLD-associated gut microbial dysbiosis [33,34].

In the present study, few significant interactions were identified for the association between plasma calprotectin levels and suspected MAFLD, consisting of associations stratified by BMI (using a cut-off of 25 kg/m^2^) and the presence of hypertension. Plasma calprotectin levels were more strongly associated with suspected MAFLD in subjects characterized by a relatively lower BMI and by the absence of hypertension. Although we cannot definitely explain these findings, it seems plausible that systemic levels of calprotectin show less variation in the presence of either obesity or hypertension and lose predictive capacity, since calprotectin may be a marker of neutrophil involvement in the early pathogenic stages of cardiovascular diseases [18]. Concerning BMI, we should however cautiously interpret this finding, since it is also part of the FLI equation. Calprotectin is also heavily involved in the development of atherosclerosis mainly via inflammatory processes [35]. Preclinical evidence suggests that calprotectin may not only reflect neutrophilic inflammation but may also be directly involved in atherosclerosis through activating the vascular endothelium, actively recruiting neutrophils and inflammatory monocytes, and interacting with the receptor for advanced glycation end-products (RAGE), triggering inflammatory and thrombotic responses and further aggravating atherosclerosis [36,37,38]. Obviously, however, the precise value of plasma calprotectin levels across clinically relevant subgroups warrants further study since in many cases subgroup sizes were relatively unbalanced.

The elevated risk of all-cause mortality in subjects with MAFLD compared to those without is well-established and reported in many previous studies [3,8,39,40,41]. As such, the identification of predictive biomarkers for early detection and/or prediction of MAFLD development is important. In the present study, we demonstrated a significant predictive association between plasma calprotectin levels and the risk of all-cause mortality when adjusting for age, sex, current smoking, alcohol use, history of cardiovascular disease, diabetes, and hypertension. However, this association lost significance after additional adjustment for the renal function, hs-CRP, HOMA-IR and total cholesterol levels. When stratifying this analysis by suspected MAFLD (FLI ≥ 60), this association was highly significant and robust in subjects without suspected MAFLD (FLI < 60), while it remarkably disappeared in subjects with suspected MAFLD (FLI ≥ 60). This difference could potentially be explained by other established risk factors for all-cause mortality compromising the predictive value of plasma calprotectin levels in the presence of suspected MAFLD, such as manifested organ damage due to MAFLD and other comorbidities as well as persistent systemic inflammation. The latter may skew the association between calprotectin and all-cause mortality, whereas it may be a more sensitive biomarker in the presence of preclinical low-grade systemic inflammation. Altogether, plasma calprotectin levels could be a potential predictor for all-cause mortality, but our data suggest that it would be more useful in preclinical stages instead of already established disease. However, it is of utmost importance to further validate these associations, and also examine plasma calprotectin levels in relation to various disease stages of MAFLD.

The relevant strengths of this study include the relatively large sample size (over 5000 individuals), the extensive phenotypic characterization of the study cohort, and the relatively long follow-up duration of almost 10 years, which enabled the prospective assessment of the diagnostic and prognostic value of plasma calprotectin levels with regard to suspected MAFLD and the risk of all-cause mortality in a population-based setting. The well-documented nature of the study allowed us to reliably establish such associations since we were able to control for a variety of relevant potential confounding factors in the analyses. Concomitantly, several limitations of the study also warrant recognition. First of all, the cross-sectional, observational nature of the study limited causal inference from the association between plasma calprotectin levels and suspected MAFLD, so we cannot exclude the possibility of reverse causation. Furthermore, the presented results are based on a single determination of plasma calprotectin levels, whereas this does not acknowledge the potential dynamics of this protein across participants, which would require longitudinal studies. Second, the generalizability of our results is limited, given the fact that the present study population was largely of Caucasian descent and without much geographical variation since all participants inhabited the northern parts of the Netherlands. Third, elevated FLI and HSI scores were used as a proxy for suspected MAFLD in this study, whereas these scores do not directly translate into measures of hepatic accumulation of lipids, which might subsequently have led to over- or underestimation of the presence of MAFLD. Irrespective of their limitations, both FLI and HSI scores are considered to confer sufficient diagnostic accuracy for suspecting MAFLD, and their use is recommended by international guidelines for the assessment of MAFLD when evaluating MAFLD in epidemiological study of large cohorts [5]. Fourth, using FLI and HSI scores, we could not differentiate between different disease stages, e.g., between simple steatosis and hepatic fibrosis. In this respect, since the present cohort lacked data on platelet counts, we were unfortunately not able to calculate fibrosis scores such as the NAFLD fibrosis score or FIB-4. Fifth, there were neither additional blood samples available nor fecal samples to determine other calprotectin-related or neutrophil-derived biomarkers, which could have been valuable to sustain the present results and to comparatively analyze the utility with regard to the presence of MAFLD. Finally, we were unable to include all participants from the PREVEND study into the analyses, since from a number of participants no serum samples (or insufficient sample volume) were available anymore to determine plasma calprotectin levels or the necessary parameters for calculating the FLI and HSI scores.

## 4. Materials and Methods

### 4.1. Study Population and Study Design

This study was performed within the Prevention of REnal and Vascular ENd-stage Disease (PREVEND) cohort study. The PREVEND study is a prospective, population-based cohort study initiated in 1997 in the city of Groningen, the Netherlands, aiming to investigate the role of urinary albumin excretion in the development of cardiovascular and renal diseases [42]. The PREVEND study features data on a myriad of health parameters relevant to cardiovascular and renal diseases from the inhabitants of Groningen. In the period of 1997–1998, many inhabitants aged 28–75 years (*n* = 85,421) were asked to participate in the study. When doing so, they were asked to provide a first morning urine sample and to complete a postal questionnaire asking on demographics and history of cardiovascular diseases. In total, 40,856 individuals responded to this questionnaire (47.8%) and provided a urine sample. Of these individuals, participants with urinary albumin concentrations ≥10 mg/L (*n* = 7786) and a randomly selected control group with urinary albumin concentrations <10 mg/L (*n* = 3395) were invited to participate in a screening visit which was conducted at the research clinic of the University Medical Center Groningen (UMCG). Exclusion criteria were pregnancy, type 1 diabetes mellitus or insulin-treated type 2 diabetes mellitus. This study screening visit was eventually completed by 8592 individuals, of which *n* = 6000 had urinary albumin concentrations ≥10 mg/L and *n* = 2592 < 10 mg/L, and these individuals were included in follow-up investigations. In the period of 2001–2003, a second round of study screening and sample collections was initiated to collect additional information from a total of 6894 participants, and this time-point was used as baseline for the current study. From these participants, we were forced to exclude 1448 individuals of which data on clinical and biochemical variables to calculate the Fatty Liver Index (FLI) or on plasma calprotectin levels (due to either missing samples or insufficient sample volume for measurements) were not available, leading to the inclusion of a total of 5446 participants for this study. The study follow-up ended on 1 January 2011. The PREVEND study was approved by the Institutional Review Board (IRB) of the UMCG (full name in Dutch: “Medisch Ethische Toetsingscommissie”, abbreviated as “METc”, IRB no. 01/139). All participants provided written informed consent for their participation. The study was conducted in accordance with the principles of the Declaration of Helsinki (2013). The reporting of the current study conforms to the EQUATOR guideline: the Strengthening the Reporting of Observational Studies in Epidemiology (STROBE) [43].

### 4.2. Data Collection

A questionnaire asking information about demographic variables; medical history (e.g., history of cardiovascular diseases, hypertension, and diabetes); lifestyle habits (e.g., smoking and alcohol consumption); and medication use was completed by all participants. Anthropometric measurements were performed, including height (m), body weight (kg), body-mass index (BMI, defined as body weight divided by squared height, kg/m^2^), waist circumference (measured on the bare skin at the natural indentation between the 10th rib and the iliac crest), and the waist/hip ratio (waist circumference divided by the largest girth between waist and thigh) [44]. Blood pressure was measured automatically for eight minutes in supine position (Dinamap XL Model 9300 series device, Johnson & Johnson Medical, Tampa Bay, FL, USA), and the ultimate blood pressure was defined as the average of the final two measurements taken. Smoking behavior was dichotomized as “current” or “never or former” smoker. Alcohol use was documented while assuming one alcoholic drink to contain 10 g of alcohol, and was categorized as no alcohol consumption, average of 1–4 drinks per month, 2–7 drinks per week, 1–3 drinks per day, and 4 or more drinks per day. Alcohol use was incorporated as dichotomized variable in statistical analyses. A history of cardiovascular disease included the following conditions: hospitalization for myocardial ischemia, obstructive coronary artery disease, or revascularization procedures. Medication use was self-reported but combined with information from a pharmacy-dispensing registry, which has complete information on drug usage of >95% of subjects participating in the PREVEND study [44,45].

### 4.3. Study Outcomes and Definitions

The Fatty Liver Index (FLI) score algorithm was used as a proxy for suspected MAFLD [5] and was calculated according to the following formula: FLI = [e(0.953 × loge (triglycerides) + 0.139 × BMI + 0.718 × loge (GGT) + 0.053 × waist circumference − 15.745)] / [1 + e(0.953) × loge (triglycerides) + 0.139 × BMI + 0.718 × loge (GGT) + 0.053 × waist circumference − 15.745)] × 100. The optimal cut-off value of the FLI for detecting suspected MAFLD has been set as a score of 60, which has a corresponding sensitivity of 61%, specificity of 86% and accuracy of 84% as determined by ultrasonography [5]. Considering this, FLI ≥ 60 was used as a definition for suspected MAFLD in the present study, which is still one of the best-validated hepatic steatosis scores for large-scale epidemiological studies [46]. In addition to the FLI, we used the hepatic steatosis index (HSI), which is also a commonly used steatosis score but more tailored to predominantly Asian populations [4]. The HSI was calculated as: HSI = 8 × ALT/AST ratio + BMI (+2, if diabetes; +2, if female). The optimal cut-off value of the HSI for detecting suspected MAFLD is a score of 36. In the equations of FLI and HSI scores, BMI is expressed as kg/m^2^; triglycerides as mmol/L; and gamma-glutamyltransferase (GGT), alanine aminotransferase (ALT) and aspartate aminotransferase (AST) as U/L.

The estimated glomerular filtration rate (eGFR) was calculated with the combined creatinine cystatin C-based Chronic Kidney Disease Epidemiology Collaboration (CKD-EPI) equation [47]. Type 2 diabetes (T2D) was defined as a fasting glucose level ≥7.0 mmol/L, a random glucose level ≥11.1 mmol/L, a self-reported physician’s diagnosis or the use of oral antidiabetics according to the guidelines of the American Diabetic Association (ADA). Metabolic syndrome (MetS) was defined according to the revised National Cholesterol Education Program Adult Treatment Panel (NCEP-ATP) II criteria. Participants were assigned to have MetS when at least three of the following five criteria were fulfilled: (a) waist circumference >102 cm for men and >88 cm for women; (b) plasma triglycerides ≥1.7 mmol/L; (c) HDL-cholesterol <1.0 mmol/L for men and <1.3 mmol/L for women; (d) hypertension (blood pressure ≥130/85 mmHg or the use of antihypertensive drugs); and (e) hyperglycemia (fasting glucose ≥5.6 mmol/L or the use of glucose-lowering drugs). The Homeostasis Model Assessment of Insulin Resistance (HOMA-IR) was calculated as: fasting plasma insulin (mU/L) × fasting plasma glucose (mmol/L)/22.5. Information on all-cause mortality was derived from the Dutch national registry of hospital discharge diagnoses (Prismant). This information was classified according to the International Statistical Classification of Diseases (ICD-10), as well as the International Classification of Health Interventions [48].

### 4.4. Laboratory Measurements

Fasting venous blood samples were obtained during the second visit, after an overnight fast, while participants had rested for 15 min. Serum and heparinized plasma samples were collected by centrifugation at 1400× *g* for 15 min at −4 °C. Plasma samples were stored at −80 °C until further analysis. Similarly, first void urine samples were collected, as well as 24-h urine samples for two consecutive days after participants were provided with both oral and written instructions, and these samples were stored at −20 °C until analysis. Urinary albumin excretion (UAE) was measured by nephelometry (Dade Behring Diagnostics, Marburg, Germany), and the average of two measurements performed in two separate 24-h urine collections was taken for analyses. Serum cystatin C (Gentian Cystatin C Immunoassay, Gentian AS, Moss, Norway) was measured on a modular analyzer (Roche Diagnostics, Roche, Basel, Switzerland). Standards were used for calibrating cystatin C according to manufacturer’s instructions and following the guidelines of the International Federation of Clinical Chemistry Working Group for Standardization of Serum Cystatin C [49]. High-sensitive C-reactive protein (hs-CRP) was measured using nephelometry (Dade Behring Diagnostics, Marburg, Germany). Serum aspartate aminotransferase (AST), alanine aminotransferase (ALT), and alkaline phosphatase (ALP) were measured using a standardized kinetic method with pyridoxal phosphate activation (Roche Modular P, Roche Diagnostics, Mannheim, Germany). Serum gamma-glutamyltransferase (GGT) was measured enzymatically using a colorimetric method (Roche Modular P, Roche Diagnostics, Mannheim, Germany). Standardization of AST, ALT, ALP and GGT was performed according to the guidelines of the International Federation of Clinical Chemistry [50,51,52,53]. The serum total cholesterol and glucose levels were measured using dry chemistry (Eastman Kodak). Low-density lipoprotein (LDL) cholesterol levels were determined by the Friedewald formula (if triglycerides were ≤4.5 mmol/L). High-density lipoprotein (HDL) cholesterol was measured using a homogeneous method (direct HDL, AerosetTM System, Abbott Laboratories, Chicago, IL, USA). Triglycerides were measured using an enzymatic method. Serum insulin was measured using a luminescence-based immunoassay. Plasma calprotectin levels were determined using the Gentian Calprotectin turbidimetric immunoassay (Gentian, Moss, Norway), which was applied on a Mindray BS-400 analyzer (Mindray, Shenzhen, China).

### 4.5. Statistical Analyses

Baseline demographic, clinical and laboratory characteristics of study participants were presented as means ± standard deviations (SD), medians with [interquartile range, IQR] in case of non-normal distributions, or as proportions *n* with corresponding percentages (%). Assessment of normality was performed by visual inspection of normal probability (Q-Q) plots and histograms. Differences in characteristics between groups were tested using independent-sample *t*-tests, Mann–Whitney *U* tests, one-way analysis of variance (ANOVA), or Kruskal–Wallis tests and chi-square tests, where appropriate. Plasma calprotectin levels were ^2^log-transformed before entry into analysis to facilitate results interpretation (expressed as per doubling). Univariable and multivariable logistic regression analyses were performed to study associations between plasma calprotectin levels and suspected MAFLD, and the results were expressed as odds ratios (ORs) (per doubling) with corresponding 95% confidence intervals (CI). Subsequently, stratified analyses were performed to study these associations across various relevant subgroups and to test for potential interactions for covariates by fitting models containing interaction terms (where *P*_interaction_ < 0.05 was considered to indicate significant effect modification). Univariable and multivariable Cox proportional hazards regression analyses were performed to assess associations between plasma calprotectin levels and the risk of mortality, expressed as hazard ratios (HRs) (per doubling) with corresponding 95% CIs. For each predictive factor, the proportionality of hazards assumption was verified in order to confirm absence of violation of model assumptions. Additionally, restricted cubic splines (RCS) were fitted containing three knots in order to evaluate potential nonlinearity of the associations observed in Cox proportional hazards regression models. Nonlinearity was assessed using likelihood ratio tests in which nested models were compared using linear or linear and cubic spline terms. Survival distributions for participants with and without suspected MAFLD were evaluated according to tertiles of plasma calprotectin levels using Kaplan–Meier curves, which were compared to each other using log-rank tests. Survival time was defined from baseline (time of sample collection) until the date of the last examination that participants attended, either death or 1 January 2011 (end of follow-up). Data analysis and visualization was performed using SPSS Statistics 28.0 software package (SPSS Inc, Chicago, IL, USA), R version 3.5.2 (Vienna, Austria), and the Python programming language (v.3.8.6, Python Software Foundation, https://www.python.org), using the *pandas* (v.1.2.3), *numpy* (v.1.20.0), *matplotlib* (v.3.4.1), *seaborn* (v.0.11.1), and *zepid* (v.0.9.0) packages. Two-tailed *p*-values ≤0.05 were considered statistically significant.

## 5. Conclusions

In conclusion, plasma calprotectin levels are significantly elevated in subjects with suspected MAFLD, even after adjustment for a large number of well-established risk factors for MAFLD. Furthermore, plasma calprotectin levels were significantly associated with the risk of all-cause mortality in the general population, although particularly in subjects with a low probability of having suspected MAFLD (FLI < 60) and not in subjects with suspected MALFD (FLI ≥ 60). Future studies are warranted to establish the definitive role and clinical utility of plasma calprotectin as a potential biomarker for suspected MAFLD, including an assessment in relation to other indices of hepatic steatosis, fibrosis, as well as associations with therapeutic outcome and the risk of developing cirrhosis and related disease complications.

## Figures and Tables

**Table 1:** Baseline demographic, clinical and laboratory characteristics compared between 3854 subjects with a fatty liver index (FLI)< 60 and 1592 subjects with a FLI ≥ 60.

	FLI < 60	FLI ≥ 60	*p*-Value
	*n* = 3854	*n* = 1592	
**Demographics**			
Age (years)	49.8 [42.1–59.4]	56.0 [47.9–65.8]	<0.001
Sex			<0.001
*Male, n (%)*	1570 (40.7)	1066 (67.0)	
*Female, n (%)*	2284 (59.3)	526 (33.0)	
Ethnicity			0.233
*White, n (%)*	3676 (96.1)	1520 (96.1)	
*Black, n (%)*	28 (0.7)	19 (1.2)	
*Asian, n (%)*	82 (2.1)	26 (1.6)	
*Other, n (%)*	41 (1.1)	16 (1.0)	
**Anthropometrics**			
BMI, kg/m^2^	24.6 [22.8–26.7]	30.1 [28.0–32.9]	<0.001
Waist circumference (cm)	86 [79–93]	104 [99–110]	<0.001
Waist/hip ratio	0.87 [0.82–0.92]	0.97 [0.91–1.01]	<0.001
**Cardiovascular risk factors**			
Current smokers, *n* (%)	1076 (28.2)	423 (26.9)	0.303
Alcohol consumption, *n* (%)	2930 (76.7)	1158 (73.2)	0.006
*None, n (%)*	891 (67.7)	425 (32.3)	
*1–4 drinks per month, n (%)*	657 (72.2)	253 (27.8)	
*2–7 drinks per week, n (%)*	1289 (74.0)	453 (26.0)	
*1–3 drinks per day, n (%)*	856 (71.2)	347 (28.8)	
*4 or more drinks per day, n (%)*	128 (54.9)	105 (45.1)	
SBP (mmHg)	118 [109–131]	133 [123–144]	<0.001
DBP (mmHg)	71 [65–77]	77 [71–83]	<0.001
Heart rate (bpm)	67 [61–74]	69 [63–76]	<0.001
**Comorbidities**			
History of cardiovascular disease, *n* (%)	96 (2.5)	84 (5.3)	<0.001
History of diabetes, *n* (%)	47 (1.2)	82 (5.2)	<0.001
Metabolic syndrome, *n* (%)	1195 (31.0)	933 (58.6)	<0.001
**Medication usage**			
Antihypertensive medication, *n* (%)	491 (12.7)	496 (31.2)	<0.001
Lipid-lowering drugs, *n* (%)	193 (5.0)	191 (12.0)	<0.001
Oral glucose-lowering drugs, *n* (%)	24 (0.7)	32 (4.5)	<0.001
**Laboratory parameters**			
Glucose (mmol/L)	4.7 [4.4–5.1]	5.1 [4.6–5.6]	<0.001
Insulin (mU/L)	6.8 [5.1–9.2]	12.6 [9.4–18.2]	<0.001
HOMA-IR (mU × mmol/L^2^/22.5)	1.43 [1.04–2.00]	2.87 [2.04–4.26]	<0.001
UAE (mg/24 h)	7.8 [5.7–12.5]	10.8 [7.1–23.1]	<0.001
hs-CRP (mg/L)	1.02 [0.49–2.27]	2.33 [1.17–4.19]	<0.001
eGFR (mL/min/1.73 m^2^)	96.3 [84.9–106.1]	89.3 [77.4–100.6]	<0.001
Cystatin C (mg/dL)	0.85 [0.77–0.95]	0.93 [0.84–1.04]	<0.001
AST (U/L)	21 [19–25]	25 [21–30]	<0.001
ALT (U/L)	15 [12–20]	23 [17–32]	<0.001
ALP (U/L)	63 [52–75]	72 [61–85]	<0.001
GGT (U/L)	19 [14–27]	41 [29–62]	<0.001
Total cholesterol (mmol/L)	5.25 [4.63–5.98]	5.76 [5.08–6.45]	<0.001
LDL-cholesterol (mmol/L)	3.44 [2.87–4.08]	3.78 [3.19–4.42]	<0.001
HDL-cholesterol (mmol/L)	1.30 [1.12–1.52]	1.06 [0.92–1.23]	<0.001
Triglycerides (mmol/L)	0.94 [0.71–1.25]	1.73 [1.29–2.34]	<0.001
Plasma calprotectin (mg/L)	0.46 [0.34–0.65]	0.57 [0.42–0.79]	<0.001

Abbreviations: ALT, alanine aminotransferase; AST, aspartate aminotransferase; BMI, body-mass index; CHF, chronic heart failure; CKD, chronic kidney disease; CVD, cardiovascular disease; DBP, diastolic blood pressure; eGFR, estimated glomerular filtration rate; FLI, fatty liver index; GGT, gamma-glutamyltransferase; HDL, high-density lipoprotein; HOMA-IR, Homeostatic Model Assessment for Insulin Resistance; hs-CRP, high-sensitive C-reactive protein; LDL, low-density lipoprotein; SBP, systolic blood pressure; UAE, urinary albumin excretion.

**Table 2:** Univariable and multivariable logistic regression analyses investigating the association between the FLI and plasma calprotectin levels (^2^log-transformed).

	Model 1		Model 2		Model 3		Model 4	
	OR (95% CI)	*p*-Value	OR (95% CI)	*p*-Value	OR (95% CI)	*p*-Value	OR (95% CI)	*p*-Value
Plasma calprotectin (2-log)	1.68 (1.56–1.82)	<0.001	1.58 (1.45–1.71)	<0.001	1.51 (1.38–1.64)	<0.001	1.19 (1.06–1.33)	0.003
Age			1.03 (1.03–1.04)	<0.001	1.01 (1.00–1.02)	<0.001	0.99 (0.98–1.00)	0.005
Sex (ref = male)			0.36 (0.32–0.41)	<0.001	0.34 (0.30–0.39)	<0.001	0.28 (0.23–0.33)	<0.001
Diabetes (ref = no)					3.01 (2.02–4.47)	<0.001	0.78 (0.44–1.40)	0.410
History of CVD (ref = no)					0.96 (0.69–1.35)	0.822	0.98 (0.65–1.49)	0.928
Hypertension (ref = no)					2.68 (2.31–3.11)	<0.001	1.95 (1.63–2.34)	<0.001
Current smoking (ref = no)					0.94 (0.81–1.09)	0.404	1.01 (0.85–1.21)	0.892
Alcohol use (ref = no)					0.78 (0.67–0.90)	0.001	0.94 (0.78–1.14)	0.544
Total cholesterol							1.53 (1.41–1.65)	<0.001
eGFR							0.99 (0.98–0.99)	<0.001
HOMA-IR							2.22 (2.07–2.38)	<0.001
hs-CRP							1.03 (1.02–1.05)	<0.001

Abbreviations: CI, confidence interval; CRP, C-reactive protein; CVD, cardiovascular disease; eGFR, estimated glomerular filtration rate; HOMA-IR, Homeostatic Model Assessment for Insulin Resistance; OR, odds ratio.

**Table 3:** Cox proportional hazards regression analyses for associations between plasma calprotectin levels and the risk of all-cause mortality in the total cohort (A) and separately in subjects with FLI < 60 (B) and FLI ≥ 60 (C).

	HR per Doubling	T1	T2	T3
		<0.41 mg/L	0.41–0.61 mg/L	>0.61 mg/L
**A. Total cohort** (*n* = 5446)				
Model 1	1.48 (1.28–1.71), ***p* < 0.001**	1.00 (reference)	1.25 (0.91–1.70), *p* = 0.172	1.73 (1.29–2.32), ***p* < 0.001**
Model 2	1.28 (1.10–1.50), ***p* = 0.002**	1.00 (reference)	1.08 (0.79–1.48), *p* = 0.632	1.24 (0.92–1.66), *p* = 0.155
Model 3	1.17 (1.00–1.38), ***p* = 0.049**	1.00 (reference)	1.00 (0.73–1.38), *p* = 0.982	1.07 (0.80–1.45), *p* = 0.643
Model 4	1.10 (0.91–1.33), *p* = 0.348	1.00 (reference)	0.93 (0.66–1.31), *p* = 0.685	0.89 (0.64–1.24), *p* = 0.498
**B. FLI < 60** (*n* = 3854)				
Model 1	1.66 (1.37–2.00), ***p* < 0.001**	1.00 (reference)	1.42 (0.94–2.15), *p* = 0.095	2.10 (1.42–3.10), ***p* < 0.001**
Model 2	1.44 (1.18–1.77), ***p* < 0.001**	1.00 (reference)	1.16 (0.77–1.75), *p* = 0.492	1.41 (0.95–2.09), *p* = 0.088
Model 3	1.33 (1.07–1.65), ***p* = 0.010**	1.00 (reference)	1.03 (0.68–1.56), *p* = 0.895	1.22 (0.81–1.81), *p* = 0.341
Model 4	1.34 (1.05–1.72), ***p* = 0.019**	1.00 (reference)	1.04 (0.67–1.63), *p* = 0.863	1.18 (0.76–1.83), *p* = 0.463
**C. FLI ≥ 60** (*n* = 1592)				
Model 1	1.07 (0.84–1.36), *p* = 0.604	1.00 (reference)	0.83 (0.51–1.34), *p* = 0.435	0.97 (0.62–1.50), *p* = 0.875
Model 2	1.07 (0.84–1.35), *p* = 0.598	1.00 (reference)	0.99 (0.61–1.61), *p* = 0.967	1.02 (0.65–1.58), *p* = 0.947
Model 3	1.01 (0.80–1.28), *p* = 0.942	1.00 (reference)	1.03 (0.63–1.69), *p* = 0.892	0.95 (0.61–1.49), *p* = 0.815
Model 4	0.88 (0.64–1.20), *p* = 0.406	1.00 (reference)	0.90 (0.53–1.53), *p* = 0.686	0.70 (0.42–1.17), *p* = 0.170

Model 1, crude model. Model 2, model 1 + with adjustment for age and sex. Model 3, model 2 with adjustment for current smoking, alcohol consumption (dichotomized), history of cardiovascular diseases, history of diabetes, and hypertension. Model 4, model 3 with additional adjustment for high-sensitive C-reactive protein (hs-CRP), insulin resistance (HOMA-IR), estimated glomerular filtration rate (eGFR), and total cholesterol levels. **Bold** *p*-values indicate statistical significance. Abbreviations: FLI, fatty liver index; HR, hazard ratio.

## Data Availability

The datasets generated for this study are available on reasonable request to the corresponding author.

## Supplementary Materials

The supporting information can be downloaded at: https://www.mdpi.com/article/10.3390/ijms232415708/s1.

## References

[1] Eslam M., Newsome P.N., Sarin S.K., Anstee Q.M., Targher G., Romero-Gomez M., Zelber-Sagi S., Wong V.W.-S., Dufour J.-F., Schattenberg J.M. (2020). A new definition for metabolic dysfunction-associated fatty liver disease: An international expert consensus statement. J. Hepatol..

[2] Loomba R., Sanyal A.J. (2013). The global NAFLD epidemic. Nat. Rev. Gastroenterol. Hepatol..

[3] van den Berg E.H., Amini M., Schreuder T.C.M.A., Dullaart R.P.F., Faber K.N., Alizadeh B.Z., Blokzijl H. (2017). Prevalence and determinants of non-alcoholic fatty liver disease in lifelines: A large Dutch population cohort. PLoS ONE.

[4] Lee J.H., Kim D., Kim H.J., Lee C.H., Yang J.I., Kim W., Kim Y.J., Yoon J.-H., Cho S.-H., Sung M.-W. (2010). Hepatic steatosis index: A simple screening tool reflecting nonalcoholic fatty liver disease. Dig. Liver Dis..

[5] Bedogni G., Bellentani S., Miglioli L., Masutti F., Passalacqua M., Castiglione A., Tiribelli C. (2006). The Fatty Liver Index: A simple and accurate predictor of hepatic steatosis in the general population. BMC Gastroenterol..

[6] Asrih M., Jornayvaz F.R. (2013). Inflammation as a potential link between nonalcoholic fatty liver disease and insulin resistance. J. Endocrinol..

[7] Gambino R., Musso G., Cassader M. (2011). Redox balance in the pathogenesis of nonalcoholic fatty liver disease: Mechanisms and therapeutic opportunities. Antioxid. Redox Signal..

[8] Damba T., Bourgonje A.R., Abdulle A.E., Pasch A., Sydor S., van den Berg E.H., Gansevoort R.T., Bakker S.J.L., Blokzijl H., Dullaart R.P.F. (2020). Oxidative stress is associated with suspected non-alcoholic fatty liver disease and all-cause mortality in the general population. Liver Int..

[9] Liu K., Wang F.S., Xu R. (2021). Neutrophils in liver diseases: Pathogenesis and therapeutic targets. Cell. Mol. Immunol..

[10] Khoury T., Mari A., Nseir W., Kadah A., Sbeit W., Mahamid M. (2019). Neutrophil-to-lymphocyte ratio is independently associated with inflammatory activity and fibrosis grade in nonalcoholic fatty liver disease. Eur. J. Gastroenterol. Hepatol..

[11] van der Windt D.J., Sud V., Zhang H., Varley P.R., Goswami J., Yazdani H.O., Tohme S., Loughran P., O’Doherty R.M., Minervini M.I. (2018). Neutrophil extracellular traps promote inflammation and development of hepatocellular carcinoma in nonalcoholic steatohepatitis. Hepatology.

[12] Mirea A.M., Toonen E.J.M., van den Munckhof I., Munsterman I.D., Tjwa E.T.T.L., Jaeger M., Oosting M., Schraa K., Rutten J.H.W., van der Graaf M. (2019). Increased proteinase 3 and neutrophil elastase plasma concentrations are associated with non-alcoholic fatty liver disease (NAFLD) and type 2 diabetes. Mol. Med..

[13] Foell D., Wittkowski H., Roth J. (2009). Monitoring disease activity by stool analyses: From occult blood to molecular markers of intestinal inflammation and damage. Gut.

[14] Wang S., Song R., Wang Z., Jing Z., Wang S., Ma J. (2018). S100A8/A9 in Inflammation. Front. Immunol..

[15] Teng T.S., Ji A.L., Ji X.Y., Li Y.Z. (2017). Neutrophils and Immunity: From Bactericidal Action to Being Conquered. J. Immunol. Res..

[16] de Seny D., Fillet M., Ribbens C., Marée R., Meuwis M.A., Lutteri L., Chapelle J.-P., Wehenkel L., Louis E., Merville M.P. (2008). Monomeric calgranulins measured by SELDI-TOF mass spectrometry and calprotectin measured by ELISA as biomarkers in arthritis. Clin. Chem..

[17] Ho G.T., Lee H.M., Brydon G., Ting T., Hare N., Drummond H., Shand A.G., Bartolo D.C., Wilson R.G., Dunlop M.G. (2009). Fecal calprotectin predicts the clinical course of acute severe ulcerative colitis. Am. J. Gastroenterol..

[18] Kunutsor S.K., Flores-Guerrero J.L., Kieneker L.M., Nilsen T., Hidden C., Sundrehagen E., Seidu S., Dullaart R.P., Bakker S.J. (2018). Plasma calprotectin and risk of cardiovascular disease: Findings from the PREVEND prospective cohort study. Atherosclerosis.

[19] Bourgonje A.R., von Martels J.Z.H., de Vos P., Faber K.N., Dijkstra G. (2018). Increased fecal calprotectin levels in Crohn’s disease correlate with elevated serum Th1- and Th17-associated cytokines. PLoS ONE.

[20] Bıçakçı E., Demirtaş C.O., Çelikel Ç., Haklar G., Duman D.G. (2020). Myeloperoxidase and calprotectin; Any role as non-invasive markers for the prediction of inflammation and fibrosis in non-alcoholic steatohepatitis. Turk. J. Gastroenterol..

[21] Cai Q., Zhu J., Cui X., Xia Y., Gao H., Wang X., Cheng M. (2022). S100A9 promotes inflammatory response in diabetic nonalcoholic fatty liver disease. Biochem. Biophys. Res. Commun..

[22] Liu X., Wang Y., Ming Y., Song Y., Zhang J., Chen X., Zeng M., Mao Y. (2015). S100A9: A Potential Biomarker for the Progression of Non-Alcoholic Fatty Liver Disease and the Diagnosis of Non-Alcoholic Steatohepatitis. PLoS ONE.

[23] Gao B., Ahmad M.F., Nagy L.E., Tsukamoto H. (2019). Inflammatory pathways in alcoholic steatohepatitis. J. Hepatol..

[24] Hessian P.A., Edgeworth J., Hogg N. (1993). MRP-8 and MRP-14, two abundant Ca(2+)-binding proteins of neutrophils and monocytes. J. Leukoc. Biol..

[25] Hwang S., Yun H., Moon S., Cho Y.E., Gao B. (2021). Role of Neutrophils in the Pathogenesis of Nonalcoholic Steatohepatitis. Front. Endocrinol..

[26] Mantovani A., Cassatella M.A., Costantini C., Jaillon S. (2011). Neutrophils in the activation and regulation of innate and adaptive immunity. Nat. Rev. Immunol..

[27] Alkhouri N., Morris-Stiff G., Campbell C., Lopez R., Tamimi T.A.-R., Yerian L., Zein N.N., Feldstein A.E. (2012). Neutrophil to lymphocyte ratio: A new marker for predicting steatohepatitis and fibrosis in patients with nonalcoholic fatty liver disease. Liver Int..

[28] Yilmaz H., Yalcin K.S., Namuslu M., Celik H.T., Sozen M., Inan O., Nadir I., Turkay C., Akcay A., Kosar A. (2015). Neutrophil-Lymphocyte Ratio (NLR) Could Be Better Predictor than C-reactive Protein (CRP) for Liver Fibrosis in Non-alcoholic Steatohepatitis (NASH). Ann. Clin. Lab. Sci..

[29] Gao B., Tsukamoto H. (2016). Inflammation in Alcoholic and Nonalcoholic Fatty Liver Disease: Friend or Foe?. Gastroenterology.

[30] Nemeth T., Mocsai A. (2016). Feedback Amplification of Neutrophil Function. Trends Immunol..

[31] Serhal R., Hilal G., Boutros G., Sidaoui J., Wardi L., Ezzeddine S., Alaaeddine N. (2015). Nonalcoholic Steatohepatitis: Involvement of the Telomerase and Proinflammatory Mediators. BioMed Res. Int..

[32] Rodrigues R.M., He Y., Hwang S., Bertola A., Mackowiak B., Ahmed Y.A., Seo W., Ma J., Wang X., Park S.H. (2022). E-Selectin-Dependent Inflammation and Lipolysis in Adipose Tissue Exacerbate Steatosis-to-NASH Progression via S100A8/9. Cell. Mol. Gastroenterol. Hepatol..

[33] Schiopu A., Cotoi O.S. (2013). S100A8 and S100A9: DAMPs at the crossroads between innate immunity, traditional risk factors, and cardiovascular disease. Mediat. Inflamm..

[34] Nacken W., Roth J., Sorg C., Kerkhoff C. (2003). S100A9/S100A8: Myeloid representatives of the S100 protein family as prominent players in innate immunity. Microsc. Res. Tech..

[35] Libby P. (2012). Inflammation in atherosclerosis. Arter. Thromb. Vasc. Biol..

[36] Vogl T., Ludwig S., Goebeler M., Strey A., Thorey I.S., Reichelt R., Foell D., Gerke V., Manitz M.P., Nacken W. (2004). MRP8 and MRP14 control microtubule reorganization during transendothelial migration of phagocytes. Blood.

[37] Hofmann M.A., Drury S., Fu C., Qu W., Taguchi A., Lu Y., Avila C., Kambham N., Bierhaus A., Nawroth P. (1999). RAGE mediates a novel proinflammatory axis: A central cell surface receptor for S100/calgranulin polypeptides. Cell.

[38] Viemann D., Barczyk K., Vogl T., Fischer U., Sunderkötter C., Schulze-Osthoff K., Roth J. (2007). MRP8/MRP14 impairs endothelial integrity and induces a caspase-dependent and -independent cell death program. Blood.

[39] Liu Y., Zhong G.C., Tan H.Y., Hao F.B., Hu J.J. (2019). Nonalcoholic fatty liver disease and mortality from all causes, cardiovascular disease, and cancer: A meta-analysis. Sci. Rep..

[40] Stefan N. (2018). Nonalcoholic Fatty Liver Disease and Mortality. Clin. Gastroenterol. Hepatol..

[41] Post A., Garcia E., van den Berg E.H., Flores-Guerrero J.L., Gruppen E.G., Groothof D., Westenbrink B.D., Connelly M.A., Bakker S.J.L., Dullaart R.P.F. (2021). Nonalcoholic fatty liver disease, circulating ketone bodies and all-cause mortality in a general population-based cohort. Eur. J. Clin. Investig..

[42] Hillege H.L., Janssen W.M., Bak A.A., Diercks G.F., Grobbee D.E., Crijns H.J.G.M., van Gilst W., de Zeeuw D., de Jong P.E. (2001). Microalbuminuria is common, also in a nondiabetic, nonhypertensive population, and an independent indicator of cardiovascular risk factors and cardiovascular morbidity. J. Intern. Med..

[43] von Elm E., Altman D.G., Egger M., Pocock S.J., Gøtzsche P.C., Vandenbroucke J.P. (2007). STROBE Initiative. The Strengthening of the Reporting of Observational Studies in Epidemiology (STROBE) statement: Guidelines for reporting observational studies. Ann. Intern. Med..

[44] Kappelle P.J.W.H., Gansevoort R.T., Hillege J.L., Wolffenbuttel B.H., Dullaart R.P., on behalf of the PREVEND study group (2011). Apolipoprotein B/A-I and total cholesterol/high-density lipoprotein cholesterol ratios both predict cardiovascular events in the general population independently of nonlipid risk factors, albuminuria and C-reactive protein. J. Intern. Med..

[45] Borggreve S.E., Hillege H.L., Wolffenbuttel B.H.R., de Jong P.E., Bakker S.J.L., van der Steege G., Van Tol A., Dullaart R.P.F. (2005). The effect of cholesteryl ester transfer protein −629C→A promoter polymorphism on high-density lipoprotein cholesterol is dependent on serum triglycerides. J. Clin. Endocrinol. Metab..

[46] European Association for the Study of the Liver (EASL), European Association for the Study of Diabetes (EASD), European Association for the Study of Obesity (EASO) (2016). EASL-EASD-EASO Clinical Practice Guidelines for the management of non-alcoholic fatty liver disease. Diabetologia.

[47] Inker L.A., Schmid C.H., Tighiouart H., Eckfeldt J.H., Feldman H.I., Greene T., Kusek J.W., Manzi J., Van Lente F., Zhang Y.L. (2012). Estimating glomerular filtration rate from serum creatinine and cystatin C. N. Engl. J. Med..

[48] WHO (2011). International Statistical Classification of Diseases and Related Health Problems, 10th revision.

[49] Grubb A., Blirup-Jensen S., Lindström V., Schmidt C., Althaus H., Zegers I., on behalf of the IFCC Working Group on Standardisation of Cystatin C (WG-SCC) (2010). First certified reference material for cystatin C in human serum ERM-DA471/IFCC. Clin. Chem. Lab. Med..

[50] Schumann G., Bonora R., Ceriotti F., Férard G., Ferrero C.A., Franck P.F., Gella F.J., Hoelzel W., Jørgensen P.J., Kanno T. (2002). IFCC primary reference procedures for the measurement of catalytic activity concentrations of enzymes at 37 degrees C. International Federation of Clinical Chemistry and Laboratory Medicine. Part 5. Reference procedure for the measurement of catalytic concentration of aspartate aminotransferase. Clin. Chem. Lab. Med..

[51] Schumann G., Bonora R., Ceriotti F., Férard G., Ferrero C.A., Franck P.F., Gella F.J., Hoelzel W., Jørgensen P.J., Kanno T. (2002). IFCC primary reference procedures for the measurement of catalytic activity concentrations of enzymes at 37 degrees C. International Federation of Clinical Chemistry and Laboratory Medicine. Part 4. Reference procedure for the measurement of catalytic concentration of alanine aminotransferase. Clin. Chem. Lab. Med..

[52] Schumann G., Bonora R., Ceriotti F., Férard G., Ferrero C.A., Franck P.F., Gella F.J., Hoelzel W., Jørgensen P.J., Kanno T. (2002). IFCC primary reference procedures for the measurement of catalytic activity concentrations of enzymes at 37 degrees C. International Federation of Clinical Chemistry and Laboratory Medicine. Part 6. Reference procedure for the measurement of catalytic concentration of gamma-glutamyltransferase. Clin. Chem. Lab. Med..

[53] Schumann G., Bonora R., Ceriotti F., Férard G., Ferrero C.A., Franck P.F., Gella F.J., Hoelzel W., Jørgensen P.J., Kanno T. (2002). IFCC primary reference procedures for the measurement of catalytic activity concentrations of enzymes at 37 degrees C. Part 1. The concept of reference procedures for the measurement of catalytic activity concentrations of enzymes. Clin. Chem. Lab. Med..

