# Supplementary material for: Plasma Calprotectin Levels Associate with Suspected Metabolic-Associated Fatty Liver Disease and All-Cause Mortality in the General Population
Source: Int J Mol Sci. 2022 Dec 11;23(24):15708. doi: 10.3390/ijms232415708 (PMC9778771; doi:10.3390/ijms232415708)
Supplement: Supplementary file 1 [file ijms-23-15708-s001.zip › ijms-2081996-supplementary.pdf]

Supplementary Data

Supplementary Table S1. Baseline demographic, clinical and laboratory characteristics compared between 4,025 subjects with a hepatic steatosis index < 36 and 1,421 subjects with a HSI ≥ 36.

	**HSI < 36**	**HSI ≥ 36**	***P*-value**
	n = 4,025	*n* = 1,421	
**Demographics**			
Age (years)	50.7 [42.7-61.0]	54.2 [46.1-63.0]	<0.001
Sex			0.394
Male, n (%)	1,962 (48.7)	674 (47.4)	
Female, n (%)	2,063 (51.3)	747 (52.6)	
Race			0.017
Caucasian, n (%)	3,847 (96.3)	1,349 (95.5)	
Negroid, n (%)	26 (0.7)	21 (1.5)	
Asian, n (%)	84 (2.1)	24 (1.7)	
Other, n (%)	39 (1.0)	18 (1.3)	
**Anthropometrics**			
BMI, kg/m^2	24.8 [22.9-26.7]	30.9 [28.9-33.4]	<0.001
Waist circumference (cm)	87 [80-94]	102 [96-110]	<0.001
Waist/hip ratio	0.88 [0.82-0.94]	0.93 [0.87-0.99]	<0.001
**Cardiovascular risk factors**			
Current smokers, *n* (%)	1,187 (29.8)	312 (22.2)	<0.001
Alcohol consumption, *n* (%)	3,122 (78.2)	966 (68.5)	<0.001
None, n (%)	871 (66.2)	445 (33.8)	
1-4 drinks per month, n (%)	660 (72.5)	250 (27.5)	
2-7 drinks per week, n (%)	1,349 (77.4)	393 (22.6)	
1-3 drinks per day, n (%)	948 (78.8)	255 (21.2)	

4 or more drinks per day, n (%)	165 (70.8)	68 (29.2)	
SBP (mmHg)	119 [110-133]	131 [120-142]	<0.001
DBP (mmHg)	71 [66-78]	75 [69-82]	<0.001
Heart rate (bpm)	67 [61-74]	69 [63-76]	<0.001
**Comorbidities**			
History of cardiovascular disease, *n* (%)	131 (3.3)	49 (3.4)	0.726
History of diabetes, *n* (%)	45 (1.1)	84 (5.9)	<0.001
Metabolic syndrome, *n* (%)	1,340 (33.3)	788 (55.5)	<0.001
**Medication usage**			
Antihypertensive medication, *n* (%)	558 (14.2)	359 (25.9)	<0.001
Lipid-lowering drugs, *n* (%)	239 (5.9)	145 (10.2)	<0.001
Oral antidiabetics, *n* (%)	25 (0.7)	44 (3.5)	<0.001
**Laboratory parameters**			
Glucose (mmol/l)	4.7 [4.4-5.1]	5.1 [4.6-5.6]	<0.001
Insulin (mU/l)	7.0 [5.2-9.6]	12.5 [9.0-18.1]	<0.001
HOMA-IR (mU x mmol/l^2/22.5)	1.47 [1.05-2.11]	2.83 [1.94-4.23]	<0.001
UAE (mg/24h)	8.1 [5.8-13.4]	9.7 [6.6-18.5]	<0.001
hs-CRP (mg/l)	1.09 [0.52-2.48]	2.09 [1.08-4.14]	<0.001
eGFR (ml/min/1.73m^2)	95.3 [83.3-105.5]	91.8 [79.9-102.1]	<0.001
Cystatin C (mg/dl)	0.86 [0.77-0.97]	0.90 [0.82-1.00]	<0.001
AST (U/l)	22 [19-25]	24 [20-29]	<0.001
ALT (U/l)	15 [12-20]	25 [18-36]	<0.001
ALP (U/l)	64 [53-76]	70 [59-84]	<0.001
GGT (U/l)	21 [14-32]	33 [22-55]	<0.001
Total cholesterol (mmol/l)	5.32 [4.69-6.06]	5.60 [4.93-6.31]	<0.001

LDL-cholesterol (mmol/l)	3.48 [2.91-4.12]	3.71 [3.11-4.35]	<0.001
HDL-cholesterol (mmol/l)	1.27 [1.08-1.49]	1.12 [0.96-1.32]	<0.001
Triglycerides (mmol/l)	1.01 [0.75-1.41]	1.40 [1.02-1.96]	<0.001
Plasma calprotectin (mg/l)	0.47 [0.34-0.67]	0.56 [0.40-0.77]	<0.001

Abbreviations: ALT, alanine aminotransferase; AST, aspartate aminotransferase; BMI, body-mass index; CHF, chronic heart failure; CKD, chronic kidney disease; CVD, cardiovascular disease; DBP, diastolic blood pressure; eGFR, estimated glomerular filtration rate; GGT, gamma-glutamyltransferase; HDL, high-density lipoprotein; HOMA-IR, Homeostatic Model Assessment for Insulin Resistance; hs-CRP, high-sensitive C-reactive protein; HSI, hepatic steatosis index; LDL, low-density lipoprotein; SBP, systolic blood pressure; UAE, urinary albumin excretion.

Supplementary Table S2. Baseline demographic, clinical and laboratory characteristics, stratified by tertiles of plasma calprotectin levels.

	Tertile 1	Tertile 2	Tertile 3	*P*-value
Calprotectin	<0.41 mg/l	0.41-0.61 mg/l	>0,61 mg/l	
**Demographics**				
Age (years)	50.1 [42.1-59.5]	51.6 [43.6-61.0]	54.2 [44.9-64.7]	<0.001
Sex				<0.001
Male, *n* (%)	784 (42.4)	882 (49.4)	970 (53.5)	
Female, *n* (%)	1,064 (57.6)	902 (50.6)	844 (46.5)	
Ethnicity				0.051
White, *n* (%)	1,737 (95.0)	1,714 (96.6)	1,745 (96.6)	
Black, *n* (%)	15 (0.8)	15 (0.8)	17 (0.9)	
Asian, *n* (%)	51 (2.8)	31 (1.7)	26 (1.4)	
Other, *n* (%)	25 (1.4)	25 (1.4)	18 (1.0)	
**Anthropometrics**				
BMI, kg/m^2	25.0 [22.8-27.9]	26.2 [23.8-28.9]	26.8 [24.4-29.7]	<0.001
Waist circumference (cm)	87 [78-96]	92 [83-100]	94 [86-102]	<0.001
Waist/hip ratio	0.87 [0.81-0.94]	0.90 [0.84-0.96]	0.91 [0.85-0.97]	<0.001
**Cardiovascular risk factors**				
Current smokers, *n* (%)	374 (20.5)	460 (26.0)	665 (37.0)	<0.001
Alcohol consumption, *n* (%)	1,417 (77.5)	1,366 (77.1)	1,305 (72.3)	<0.001
None, n (%)	412 (31.3)	405 (30.8)	499 (37.9)	
1-4 drinks per month, n (%)	314 (34.5)	305 (33.5)	291 (32.0)	
2-7 drinks per week, n (%)	603 (34.6)	595 (34.2)	544 (31.2)	
1-3 drinks per day, n (%)	437 (36.3)	392 (32.6)	374 (31.1)	

4 or more drinks per day, n (%)	63 (27.0)	74 (31.8)	96 (41.2)	
SBP (mmHg)	118 [109-131]	123 [113-136]	127 [115-140]	<0.001
DBP (mmHg)	71 [65-77]	73 [67-79]	74 [68-81]	<0.001
Heart rate (bpm)	66 [60-72]	68 [62-75]	70 [63-77]	<0.001
**Comorbidities**				
History of cardiovascular disease, *n* (%)	38 (2.1)	64 (3.6)	78 (4.3)	<0.001
History of diabetes, *n* (%)	29 (1.6)	39 (2.2)	61 (3.4)	0.001
Metabolic syndrome, *n* (%)	569 (30.8)	699 (39.2)	860 (47.4)	<0.001
**Medication usage**				
Antihypertensive medication, *n* (%)	242 (13.4)	277 (15.9)	398 (22.5)	<0.001
Lipid-lowering drugs, *n* (%)	95 (5.1)	131 (7.3)	158 (8.7)	<0.001
Oral glucose-lowering drugs, *n* (%)	16 (1.0)	21 (1.4)	32 (2.0)	0.057
**Laboratory parameters**				
Glucose (mmol/l)	4.7 [4.4-5.1]	4.7 [4.4-5.2]	4.8 [4.5-5.3]	<0.001
Insulin (mU/l)	7.0 [5.1-10.2]	8.2 [6.0-11.9]	9.1 [6.4-13.0]	<0.001
HOMA-IR (mU x mmol/l^2/22.5)	1.45 [1.03-2.22]	1.75 [1.22-2.65]	1.96 [1.32-2.99]	<0.001
UAE (mg/24h)	7.6 [5.8-11.6]	8.5 [6.0-14.8]	9.6 [6.4-19.0]	<0.001
hs-CRP (mg/l)	0.81 [0.37-1.59]	1.24 [0.66-2.61]	2.41 [1.16-4.93]	<0.001
eGFR (ml/min/1.73m^2)	97.0 [85.3-106.8]	94.4 [83.1-104.7]]	92.0 [78.2-102.1]	<0.001
Cystatin C (mg/dl)	0.84 [0.76-0.94]	0.87 [0.79-0.97]	0.91 [0.81-1.03]	<0.001
AST (U/l)	22 [19-26]	22 [19-27]	23 [20-27]	<0.001
ALT (U/l)	16 [12-22]	17 [13-25]	18 [13-24]	<0.001
ALP (U/l)	60 [50-73]	66 [55-78]	71 [61-85]	<0.001
GGT (U/l)	20 [13-31]	24 [16-38]	26 [18-42]	<0.001

Total cholesterol (mmol/l)	5.26 [4.61-6.00]	5.36 [4.75-6.16]	5.51 [4.85-6.22]	<0.001
LDL-cholesterol (mmol/l)	3.41 [2.84-4.04]	3.55 [2.98-4.21]	3.65 [2.09-4.30]	<0.001
HDL-cholesterol (mmol/l)	1.32 [1.11-1.53]	1.22 [1.04-1.42]	1.16 [1.00-1.37]	<0.001
Triglycerides (mmol/l)	0.96 [0.70-1.37]	1.10 [0.81-1.57]	1.25 [0.90-1.76]	<0.001
Fatty liver index	22.8 [9.0-52.0]	37.6 [16.3-65.6]	47.5 [23.1-74.1]	<0.001

Abbreviations: ALT, alanine aminotransferase; AST, aspartate aminotransferase; BMI, body-mass index; CHF, chronic heart failure; CKD, chronic kidney disease; CVD, cardiovascular disease; DBP, diastolic blood pressure; eGFR, estimated glomerular filtration rate; GGT, gamma-glutamyltransferase; HDL, high-density lipoprotein; HOMA-IR, Homeostatic Model Assessment for Insulin Resistance; hs-CRP, high-sensitive C-reactive protein; LDL, low-density lipoprotein; SBP, systolic blood pressure; UAE, urinary albumin excretion.

Supplementary Table S3. Univariable and multivariable logistic regression analyses investigating the association between the HSI and plasma calprotectin levels (^{2}log-transformed).

	Model 1		Model 2		Model 3		Model 4	
	OR (95% CI)	*P*-value	OR (95% CI)	*P*-value	OR (95% CI)	*P*-value	OR (95% CI)	*P*-value
Plasma calprotectin (2-log)	1.47 (1.35-1.59)	<0.001	1.45 (1.34-1.57)	<0.001	1.43 (1.32-1.56)	<0.001	1.24 (1.12-1.38)	<0.001
Age			1.01 (1.01-1.02)	<0.001	0.99 (0.99-1.00)	0.046	0.99 (0.98-0.99)	<0.001
Sex (ref=male)			1.14 (1.01-1.29)	0.039	1.10 (0.97-1.26)	0.143	1.28 (1.10-1.49)	0.001
Diabetes (ref=no)					4.65 (3.14-6.89)	<0.001	1.64 (0.97-2.79)	0.066
History of CVD (ref=no)					0.68 (0.48-0.98)	0.039	0.59 (0.38-0.92)	0.019
Hypertension (ref=no)					2.22 (1.91-2.58)	<0.001	1.61 (1.35-1.91)	<0.001
Current smoking (ref=no)					0.63 (0.54-0.73)	<0.001	0.64 (0.54-0.76)	<0.001
Alcohol use (ref=no)					0.68 (0.59-0.78)	<0.001	0.76 (0.64-0.89)	0.001
Total cholesterol							1.15 (1.07-1.23)	<0.001

eGFR							1.00 (0.99-1.01)	0.899
HOMA-IR							1.76 (1.66-1.86)	<0.001
hs-CRP							1.02 (1.00-1.03)	0.048

Abbreviations: CI, confidence interval; CRP, C-reactive protein; CVD, cardiovascular disease; eGFR, estimated glomerular filtration rate; HOMA-IR, Homeostatic Model Assessment for Insulin Resistance; OR, odds ratio.

Supplementary Table S4. Stratified analyses for the association between plasma calprotectin levels (^{2}log-transformed) and suspected MAFLD (FLI ≥60) across various subgroups.

Variable	Total (*n*)	OR	95% CI	*P*-value for interaction
**Overall**	5446	1.19	1.06-1.33	
**Gender**				
Male	2636	1.20	1.03-1.39	0.462
Female	2810	1.19	1.00-1.43	
**BMI**				
< 25 kg/m^2	2177	1.32	0.86-2.04	**<0.001**
≥ 25 kg/m^2	3257	1.14	1.01-1.30	
**Hypertension**				
No	3797	1.24	1.07-1.44	**0.006**
Yes	1646	1.09	0.91-1.31	
**History of CVD**				
No	5266	1.19	1.06-1.34	0.699
Yes	180	1.56	0.75-3.25	
**Diabetes**				
No	5305	1.18	`1.05-1.32	0.732
Yes	129	0.82	0.35-1.93	

**UAE**				
< 8.4 mg/24-h	2723	1.22	1.02-1.45	0.999
> 8.4 mg/24-h	2723	1.13	0.97-1.32	
**Alcohol consumption**				
No	1316	1.15	0.92-1.43	0.464
Yes	4088	1.21	1.06-1.38	
**Current smoking**				
No	3886	1.23	1.07-1.40	0.164
Yes	1499	1.06	0.85-1.32	
**Total cholesterol**				
< 5.38 mmol/l	2723	1.31	1.10-1.56	0.368
> 5.38 mmol/l	2723	1.09	0.93-1.26	

Abbreviations: BMI, body-mass index; CI, confidence interval; CVD, cardiovascular disease; OR, odds ratio; UAE, urinary albumin excretion.

Supplementary Table S5. Stratified analyses for the association between plasma calprotectin levels (^{2}log-transformed) and suspected MAFLD (HSI ≥36) across various subgroups.

Variable	Total (*n*)	OR	95% CI	***P*-value for interaction**
**Overall**	5446	1.24	1.12-1.38	
**Gender**				
Male	2636	1.16	0.99-1.35	**0.013**

Female	2810	1.28	1.10-1.48	
**BMI**				
< 25 kg/m^2	2177	1.43	0.82-2.50	**<0.001**
≥ 25 kg/m^2	3257	1.15	1.02-1.29	
**Hypertension**				
No	3797	1.25	1.10-1.43	**0.030**
Yes	1646	1.18	0.99-1.41	
**History of CVD**				
No	5266	1.24	1.12-1.38	0.277
Yes	180	1.19	0.60-2.39	
**Diabetes**				
No	5305	1.22	1.09-1.36	0.297
Yes	129	1.39	0.62-3.08	
**UAE**				
< 8.4 mg/24-h	2723	1.32	1.13-1.54	0.993
> 8.4 mg/24-h	2723	1.18	1.02-1.36	
**Alcohol consumption**				
No	1316	1.24	1.01-1.52	0.685

Yes	4088	1.24	1.10-1.40	
**Current smoking**				
No	3886	1.23	1.09-1.39	0.272
Yes	1499	1.24	0.99-1.55	
**Total cholesterol**				
< 5.38 mmol/l	2723	1.32	1.13-1.54	0.089
> 5.38 mmol/l	2723	1.16	1.00-1.34	

Abbreviations: BMI, body-mass index; CI, confidence interval; CVD, cardiovascular disease; OR, odds ratio; UAE, urinary albumin excretion.

Supplementary Table S6. Cox proportional hazards regression analyses for associations between plasma calprotectin levels and the risk of all-cause mortality in subjects with HSI<36 (A) and HSI ≥36 (B).

	**HR per doubling**	**T1**	**T2**	**T3**
		<0.41 mg/l	0.41-0.61 mg/l	>0,61 mg/l
**A. HSI <36** (*n* = 4,025)				
Model 1	1.57 (1.33-1.85), ***P*<0.001**	1.00 (reference)	1.13 (0.78-1.62), *P*=0.520	1.91 (1.37-2.65), ***P*<0.001**
Model 2	1.31 (1.10-1.57), ***P*=0.003**	1.00 (reference)	0.91 (0.63-1.31), *P*=0.614	1.21 (0.87-1.69), *P*=0.255
Model 3	1.20 (1.00-1.44), *P*=0.054	1.00 (reference)	0.83 (0.58-1.20), *P*=0.322	1.05 (0.75-1.47), *P*=0.794
Model 4	1.18 (0.95-1.47), P=0.143	1.00 (reference)	0.80 (0.54-1.18), *P*=0.263	0.95 (0.65-1.38), *P*=0.771
**B. HSI ≥36** (*n* = 1,421)				

Model 1	1.23 (0.89-1.69), *P*=0.215	1.00 (reference)	1.65 (0.85-3.19), *P*=0.139	1.42 (0.74-2.72), *P*=0.298
Model 2	1.21 (0.88-1.67), *P*=0.234	1.00 (reference)	1.91 (0.98-3.70), *P*=0.057	1.47 (0.76-2.83), *P*=0.248
Model 3	1.15 (0.83-1.59), *P*=0.408	1.00 (reference)	1.92 (0.98-3.73), *P*=0.056	1.38 (0.71-2.67), *P*=0.346
Model 4	0.89 (0.60-1.33), *P*=0.569	1.00 (reference)	1.44 (0.71-2.93), *P*=0.315	0.78 (0.37-1.65), *P*=0.522

Model 1, crude model. Model 2, model 1 + with adjustment for age and sex. Model 3, model 2 with adjustment for current smoking, alcohol consumption, history of cardiovascular diseases, history of diabetes, and hypertension. Model 4, model 3 with additional adjustment for high-sensitive C-reactive protein (hs-CRP), insulin resistance (HOMA-IR), estimated glomerular filtration rate (eGFR), and total cholesterol levels. **Bold** *P*-values indicate statistical significance. Abbreviations: HSI, hepatic steatosis index; HR, hazard ratio.
